# Self-esteem in women experienced intimate partner violence : a tunisian cross-sectional study

**DOI:** 10.1192/j.eurpsy.2022.2233

**Published:** 2022-09-01

**Authors:** K. Safa, S. Hentati, M. Bouhamed, B.C. Syrine, I. Baati, J. Masmoudi

**Affiliations:** Hedi Chaker University hospital, Psychiatry A, sfax, Tunisia

**Keywords:** violence-Self-esteem-women-psychological

## Abstract

**Introduction:**

Intimate partner violence (IPV) and psychological distress are major public health concerns among emerging adult women.

**Objectives:**

To study the self-esteem of women victims of domestic violence and to determine its associated factors .

**Methods:**

This was a cross-sectional descriptive study carried out at the National Health Fund of Sfax among women who consulted during the months of October and November 2019. The sociodemographic and clinical characteristics of the consultants were collected using a pre-established form. Women’s Experience with Battering Scale” (WEBS) was used to screen (IPV). The Rosenberg Self-Esteem Scale (RSE), was used for evaluating individual self-esteem. It uses a scale of 0–4 where a score less than 25 may indicate a problematic very low self esteem.

**Results:**

The sample comprised 110 women. More than half (66.7%) of women had a primary school level and 69% had a median socioeconomic level. (IPV) was estimated at 57.3% in our population. The mean (WEBS) score was 30.92(SD=9.8) and the mean (RSE) score was 31.26 (SD=3.5) among abused women self-esteem was very low in15.5% and low in 42.7% Abused women were more likely to have a low self-esteem (r= -0.528 ;p=0.012) The score of self-esteem decreased with age (r=-0.685 p=0.0001) and previous history of violence(p=0.04).

**Conclusions:**

The findings suggests that Women who experienced (IPV) were more likely to have a low self-esteem. Therefore, the role of the physician is essential not only in the care of the victims but also in the detection of psychological repercussions.

**Disclosure:**

No significant relationships.

